# On the Catarrhal Pneumonia and Lobar Pneumonia of Children

**Published:** 1853-01

**Authors:** 


From the British and Foreign Med. Chir. Rev. and Dublin Med. Press.

*On the Catarrhal Pneumonia and Lobar Pneumonia of Children, by* MM. TROUSSEAU and LASEGUE.

CATARRHAL (or lobular) pneumonia is a disease as distinct from simple (lobar) as variola is from erythema. This is seen in their respective mortality. Of twenty children who have been admitted into the hospital clinique suffering from simple pneumonia, in six months all have recovered; of nearly thirty who were attacked with catarrhal pneumonia, not one survived. Most of the first class of cases exhibited an excessive degree of acuteness, which burnt out like a fire of straw; while several of the second, notwithstanding their fatal termination, commenced with very mild symptoms.

Simple pneumonia hardly ever affects a child below two years of age, and rarely those of two or three, but becomes of more and more frequent occurrence as the child approaches adolescence. Its cause and symptoms resemble those of the adult, with some modifications. After twenty-four or thirty-six hours, the souffle and bronchophony can alone be heard; the crepitant rale, which is often observed in the adult when the patient coughs, even when souffle is present, is hardly ever heard in the child. So afterwards, from day to day, without the crepitation of resolution, the souffle disappears, leaving only a feeble respiration. The progress of the disease is also more rapid than in the adult. In the mild form of the disease, recovery takes place rapidly, and in large proportion; but in its grave form, many cases are lost by any mode of treatment. M. Trousseau generally bleeds the child, gives it an emetic of sulphate of copper, and then a mixture containing Kerme's mineral and extract of digitalis.

Catarrhal pneumonia commences with a catarrh, which rapidly extends to the small bronchi, and then we hear numerous and small subcrepitant rales disseminated over both lungs, and especially posteriorly. These rales may persist for four, six, eight, or fifteen days, without any souffle becoming manifest; but sooner or later we hear a souffle, the resonance of the cries of the voice, or at least a prolonged respiratory murmur. While these latter sounds, common to simple and catarrhal pneumonia, are thus manifesting themselves, we find by the subcrepitant rales that the capillary catarrh is still persisting in the rest of the lung. The disease has extended from the mucous membrane to the parenchyma of the organ. Febrile action is less than in ordinary pneumonia, being predominant at some portions of the day and entirely ceasing at others; and these alternations of better and worse may continue for fifteen, twenty, or thirty days; the disease being originally a pulmonary catarrh, and partaking of the obstinacy and uncertainty of catarrhal complaints. As more and more of the parenchyma becomes implicated, the fever becomes more continuous and intense, and the respiration more difficult, until the children die exhausted. In other cases, in which the bronchial phlegmasia was very intense, from the first, and the lung became rapidly invaded over a great extent, death takes place with rapidity. The progress of the disease has usually been more rapidly fatal when it has succeeded to measles, chronic disease of the skin, or laryngitis. All means of treatment that have been tried have proved impotent.

These two affections may be compared, *exceptis excipiendis*, with erysipelas and phlegmon. Erysipelas traverses the surface, like the catarrh; and when it persists too long, it induces ulcerations of the skin, furuncles, and circumscribed subcutaneous abscess, just as the capillary catarrh induces suppuration of the lobules, little abscesses of the lungs, and circumscribed pneumonias. Simple pneumonia, on the other hand, progresses like simple phlegmon, violent in its febrile reaction, but terminating abruptly and rapidly.

It must not be supposed, from what has been said, that catarrhal pneumonia is almost invariably fatal. Although this is the case amidst the miasmata of an hospital, which exert effects at once so terrible and so difficult to avert, it is not so in private practice. In this, one-half the patients may be cured by repeated vomiting, flying blisters, antimonials, and digitalis; but how terrible are the ravages of a disease which, under the most favorable circumstances, kills one-half its subjects!

